# Longitudinal Analysis of T and B Cell Phenotype and Function in Renal Transplant Recipients with or without Rituximab Induction Therapy

**DOI:** 10.1371/journal.pone.0112658

**Published:** 2014-11-13

**Authors:** Elena G. Kamburova, Hans J. P. M. Koenen, Martijn W. F. van den Hoogen, Marije C. Baas, Irma Joosten, Luuk B. Hilbrands

**Affiliations:** 1 Department of Laboratory Medicine - Medical Immunology, Radboud University Medical Centre, Nijmegen, The Netherlands; 2 Department of Nephrology, Radboud University Medical Centre, Nijmegen, The Netherlands; Public Health England, United Kingdom

## Abstract

**Background:**

Prevention of rejection after renal transplantation requires treatment with immunosuppressive drugs. Data on their in vivo effects on T- and B-cell phenotype and function are limited.

**Methods:**

In a randomized double-blind placebo-controlled study to prevent renal allograft rejection, patients were treated with tacrolimus, mycophenolate mofetil (MMF), steroids, and a single dose of rituximab or placebo during transplant surgery. In a subset of patients, we analyzed the number and phenotype of peripheral T and B cells by multiparameter flow cytometry before transplantation, and at 3, 6, 12, and 24 months after transplantation.

**Results:**

In patients treated with tacrolimus/MMF/steroids the proportion of central memory CD4^+^ and CD8^+^ T cells was higher at 3 months post-transplant compared to pre-transplant levels. In addition, the ratio between the percentage of central memory CD4^+^ and CD4^+^ regulatory T cells was significantly higher up to 24 months post-transplant compared to pre-transplant levels. Interestingly, treatment with tacrolimus/MMF/steroids resulted in a shift toward a more memory-like B-cell phenotype post-transplant. Addition of a single dose of rituximab resulted in a long-lasting B-cell depletion. At 12 months post-transplant, the small fraction of repopulated B cells consisted of a high percentage of transitional B cells. Rituximab treatment had no effect on the T-cell phenotype and function post-transplant.

**Conclusions:**

Renal transplant recipients treated with tacrolimus/MMF/steroids show an altered memory T and B-cell compartment post-transplant. Additional B-cell depletion by rituximab leads to a relative increase of transitional and memory-like B cells, without affecting T-cell phenotype and function.

**Trial Registration:**

ClinicalTrials.gov NCT00565331

## Introduction

Life-long use of immunosuppressive drugs is required to prevent rejection after renal transplantation. Nevertheless, the continuous use of immunosuppressive drugs does not preclude the development of chronic rejection, which is a major cause of long-term allograft loss [1]. T cells play an important role in the pathogenesis of rejection via the recognition of alloantigens, resulting in T-cell activation, proliferation, and differentiation into CD8^+^ cytotoxic T cells and CD4^+^ T helper cells [2]. Therefore, the most commonly used immunosuppressive drugs in transplantation are directed against T cells to inhibit these processes [3]. On the other hand, regulatory T cells are able to suppress the immune response and prevent allograft rejection [4]. The balance between memory and regulatory T cells during the course after transplantation can be used to predict renal graft rejection following the reduction of immunosuppressive therapy [5]. Next to T cells, B cells can be involved in graft rejection [6]. The presence of B-cell clusters in renal grafts during acute rejection or the presence of anti-HLA antibodies before transplantation is associated with poorer graft survival [7]–[9]. Notably, B cells can induce alloimmune responses by acting as professional antigen presenting cells, or by the production of various (pro-)inflammatory cytokines [10]. Therefore, depletion of B cells in renal transplant recipients might help to prevent allograft rejection. Current immunosuppressive regimens consisting of steroids, a calcineurin-inhibitor, and mycophenolate mofetil (MMF) inhibit B-cell function directly due to inhibition of their proliferation and indirectly via the inhibition of T-cell help. B cells can also be selectively depleted by rituximab (RTX), an anti-CD20 monoclonal antibody. RTX is successfully used in the treatment of B-cell malignancies and autoimmune disorders mediated by T and B cells [11], [12]. Although the major target of RTX-based treatment was to reduce the levels of circulating autoantibodies, additional B-cell functions may be affected, such as antigen presentation and cytokine production [13]. Furthermore induction of regulatory T cells (T_REGS_) was reported after RTX treatment in patients with lupus nephritis [14]. Therefore, next to its effect on B cells, RTX might decrease the chance of rejection after transplantation by affecting the T-cell compartment. Remarkably little is known about the effects of the currently used immunosuppressive strategies on the phenotype and function of T and B cells during the course after renal transplantation. Advancements in multiparameter flow cytometry have made it possible to analyze the effects of immunosuppressive agents on various T- and B-cell subsets in more detail. We had the opportunity to study the effects of standard immunosuppression (tacrolimus, MMF and steroids), with or without the addition of RTX induction therapy on the phenotype and function of T and B cells over time in renal transplant recipients participating in a randomized placebo-controlled trial, studying the efficacy and safety of RTX added to standard immunosuppression. To avoid bias by other immunological events as much as possible, we analyzed only Cytomegalovirus (CMV) seronegative patients who received a kidney from a CMV seronegative donor, did not experience a rejection episode, and were not treated with additional immunosuppressive drugs during the follow-up period.

## Materials and Methods

### Patients

Patients were selected from participants of a clinical trial with RTX in renal transplantation at our hospital (ClinicalTrials.gov, NCT00565331). This study investigated the effectiveness and safety of RTX for prophylaxis of acute rejection after renal transplantation. Patients were randomized between treatment with a single dose of RTX (375 mg/m^2^) or placebo during transplant surgery. Concomitant immunosuppression consisted of tacrolimus, MMF, and steroids. Patients received 100 mg of prednisolone intravenously during the first 3 days after transplantation and subsequently an oral dose of 15–25 mg/day, tapered to a maintenance dose of 0.1 mg/kg/day. Tacrolimus was started at 0.2 mg/kg/day and the dose was subsequently adjusted to achieve whole-blood trough levels of 15–20 ng/ml during the first 2 weeks, 10–15 ng/ml during weeks 3–6, and 5–10 ng/ml from week 7. MMF was administered at 1000 mg twice daily with a dose reduction to 750 mg twice daily at 2 weeks after transplantation, and discontinued after 6 months.

The current study was carried out as preplanned side study of the clinical trial. To create a homogeneous study population without disturbing immunological events, we selected patients who did not meet the primary end point of the clinical trial (biopsy-proven acute rejection). Moreover, we investigated only Cytomegalovirus (CMV) seronegative patients who received a kidney from a CMV seronegative donor (and thus did not develop CMV infection), and we excluded patients in whom the immunosuppressive treatment was changed during the follow-up period. After applying these selection criteria, all remaining patients were included: 14 patients in the group treated with tacrolimus, MMF and steroids, and 12 patients in the group additionally treated with RTX (Figure S1). Peripheral blood mononuclear cells (PBMCs) of 4 standard-treated patients and 3 RTX-treated patients were not available at 24 months after transplantation. Table 1 summarizes the characteristics of all patients. The study was approved by the Institutional review board of the Radboudumc Nijmegen. Written informed consent was obtained from all participants.

**Table 1:** Patient characteristics.

	Triple IS	Triple IS + rituximab	P
N	14	12	
Median age at Tx, years (range)	50 (20–73)	57 (25–66)	0.440
Sex, no. male (%)	11 (79%)	7 (58%)	0.401
Type of dialysis			0.446
- Hemodialysis	6	5	
- Peritoneal dialysis	2	4	
- None	6	3	
Median PRA, % (range)	0 (0–58)	0 (0–10)	0.852
Living donor, no. (%)	13 (93%)	8 (67%)	0.217
HLA mismatches total, median (range)	4 (2–6)	3 (1–5)	0.224

IS: immunosuppression.

### Cells

Peripheral blood samples were obtained before transplantation, and up to 24 months after transplantation. Whole blood counts were performed and PBMCs were isolated by density gradient centrifugation using Lymphoprep (Lucron, Dieren, The Netherlands). PBMCs were cryopreserved in liquid nitrogen until analysis. For each patient longitudinal flow cytometric analysis was performed for all available samples at the same time.

### Flow cytometry

For cell surface staining, the following fluorochrome-conjugated monoclonal antibodies were used CD3(UCHT1), CD4(13B8.2), CD8(B9.11), CD19(J3-119), CD24(ALB9), CD38(LS198-4-3), CD45(J.33), CD45RO(UCHL1), CD127(R34.34) and IgD(IADB6) (all from Beckman-Coulter, Mijdrecht, The Netherlands), CD25(M-A251), CCR4(1G1), CCR6(11A9) and CXCR3(1C6/CXR3) (BD Biosciences, Erembodegem, Belgium), and BAFF-R(11C1) (BioLegend, Uithoorn, The Netherlands). Intracellular analysis of IL-2(MQ1-17H12) (BD Biosciences), IL-4(8D4–8), IL-17(EBIO64CAP17), IFNγ(4S.B3) (eBioscience, San Diego, CA, USA), and TNFα(Mab11) (Dako, Glostrup, Denmark) was performed after fixation and permeabilization, using Fix and Perm reagent (eBioscience). Before intracellular cytokine measurement, the cells were stimulated for 4 hours with PMA (12.5 ng/ml), ionomycin (500 ng/ml) and Brefeldin A (5 µg/ml; Sigma-Aldrich, Zwijndrecht, The Netherlands).

The cell phenotype was analyzed by five-color (FC500) or ten-color flow cytometry (Navios), and data were analyzed using Kaluza software (all from Beckman-Coulter). Isotype controls or unstained cells were used for gate settings. Cell populations >0.1% of the CD45^+^ lymphocyte population with a threshold of more than 50 cells were considered reliable.

### Statistical analysis

Continuous data are expressed as box plots displaying the median, 25th and 75th percentiles as the box, and the 5th and 95th percentiles as whiskers. Wilcoxon signed rank test was used to test differences between each follow-up and pre-transplantation value within the triple immunosuppression-treated group. Statistical testing between RTX- and placebo-treated patients was performed according to distribution and type of data (unpaired T test, Mann-Whitney U, or Fisher’s exact tests). P<0.05 was considered statistically significant. Statistical analysis was performed using GraphPad Prism 5.03 (GraphPad Software Inc., La Jolla, CA, USA).

## Results

### Phenotype and function of T cells in renal transplant recipients after treatment with tacrolimus, MMF and steroids

**Figure 1:**
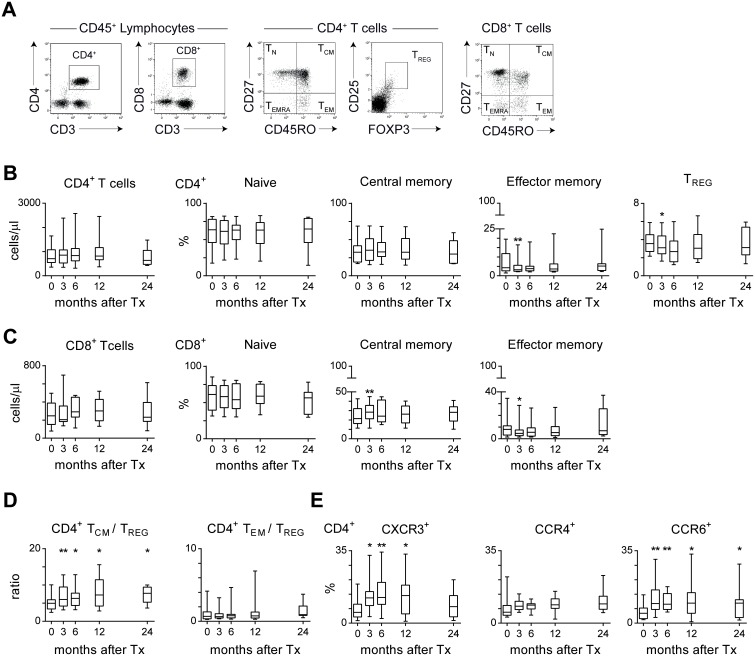
Subset distribution of circulating T cells in renal transplant recipients after treatment with tacrolimus, MMF and steroids over time. (A) Representative dot plots for a renal transplant recipient showing CD3^+^CD4^+^ and CD3^+^CD8^+^ T cells within the CD45^+^ lymphocyte population. Circulating CD4^+^ and CD8^+^ T cells can be characterized as naive (T_N_; CD27^+^CD45RO^−^), central memory (T_CM_; CD27^+^CD45RO^+^), effector memory (T_EM_; CD27^−^CD45RO^+^) and highly differentiated memory (T_EMRA_; CD27^+^CD45RO^−^) cells. Furthermore, CD4^+^ T cells can be characterized as regulatory T cells (T_REGS_; CD25^hi^FOXP3^+^). (B) Shown are the absolute numbers of CD4^+^ T cells and the percentages of T_N_, T_CM_ and T_EM_ within the CD4^+^ T-cell population for 14 triple immunosuppression-treated patients before transplantation (t = 0) and at 3, 6, 12 and 24 months after transplantation (n = 10 at 24 m). (C) As described under B, for CD8^+^ T cells. (D) The ratio between the percentage of CD4^+^ T_CM_ or T_EM_ and the percentage of T_REGS_. (E) Longitudinal analysis of the percentages of CXCR3^+^, CCR4^+^, and CCR6^+^ cells within the CD4^+^ T-cell population of 14 triple immunosuppression-treated patients (n = 10 at 24 m). Results are shown as box plots displaying the median, 25^th^ and 75^th^ percentiles as the box, and the 5^th^ and 95^th^ percentiles as whiskers. Significant differences are indicated compared to pre-transplant levels: *P<0.05, **P<0.01.

CD4^+^ and CD8^+^ T cells can be divided into naive, central and effector memory, and highly differentiated memory cells based on CD27 and CD45RO expression. [15]. The absolute numbers of CD4^+^ and CD8^+^ T cells in peripheral blood did not change during the use of triple drug immunosuppression after transplantation (Figure 1A–C). However, the percentages of central memory (T_CM_; CD27^+^CD45RO^+^) CD4^+^ and CD8^+^ T cells were significantly higher at 3 months after transplantation compared to before transplantation, while the percentages of effector memory (T_EM_; CD27^−^CD45RO^+^) CD4^+^ and CD8^+^ T cells, and regulatory (T_REGS_; CD25^hi^FOXP3^+^) CD4^+^ T cells were significantly decreased. The percentages of naive (T_N_; CD27^+^CD45RO^−^) and highly differentiated memory (T_EMRA_; CD27^−^CD45RO^−^) CD4^+^ and CD8^+^ T cells were comparable to pre-transplant levels. Next to the T-cell subset distribution, the ratio between memory and regulatory T cells might be an important determinant of the risk of rejection [16]. Interestingly, the CD4^+^ T_CM_/CD4^+^ T_REGS_ ratio significantly increased from 3 months after transplantation and remained elevated compared to the pre-transplant ratio (Figure 1D). The CD4^+^ T_EM/_CD4^+^ T_REGS_ ratio after transplantation was comparable to pre-transplant levels. Finally, we determined the expression of chemokine receptors associated with T helper (Th) 1 (CXCR3), Th2 (CCR4), and Th17 (CCR6) cells (Figure 1E). The percentage of CXCR3^+^ and CCR6^+^ CD4^+^ T cells increased during immunosuppressive treatment, whereas triple drug immunosuppression did not affect the percentage of CCR4^+^ CD4^+^ T cells.

**Figure 2:**
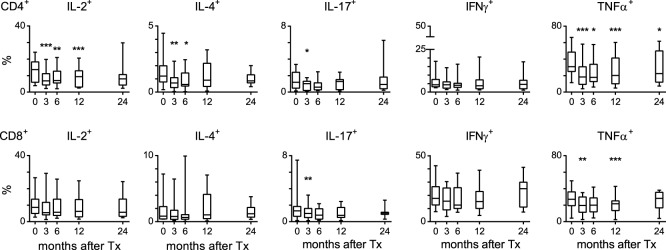
*Ex vivo* cytokine production by circulating T cells in renal transplant recipients after treatment with tacrolimus, MMF and steroids. Peripheral blood mononuclear cells (PBMCs) were stimulated for 4 hours in the presence of PMA, ionomycin and Brefeldin A. Shown are the percentages IL-2, IL-4, IL-17, IFNγ or TNFα-producing cells within the CD4^+^ or CD8^+^ T-cell population of 14 triple immunosuppression-treated patients before transplantation (t = 0) and at 3, 6, 12, and 24 months after transplantation (n = 10 at 24 m). Results are shown as box plots displaying the median, 25^th^ and 75^th^ percentiles as the box, and the 5^th^ and 95^th^ percentiles as whiskers. Significant differences are indicated compared to pre-transplant levels: *P<0.05, **P<0.01, ***P<0.001.

Although triple drug immunosuppression had minimal effects on the phenotype of peripheral T cells, this did not preclude an effect on functional capacities. To assess the cytokine producing capacity of circulating T cells, PBMCs were *ex vivo* stimulated for 4 hours with PMA, ionomycin and Brefeldin A. Effector cytokine production was assessed using intracellular cytokine staining in both CD4^+^ and CD8^+^ T cells. The percentage of IL-2, IL-4, IL-17, and TNFα producing CD4^+^ cells decreased at 3 months after transplantation compared to pre-transplant levels. Hereafter, the production of all cytokines but TNFα returned to baseline levels within 24 months after transplantation (Figure 2). *Ex vivo* IFNγ production was not affected by triple drug immunosuppression, nor was cytokine producing capacity of CD8^+^ T cells. Taken together, within the T-cell compartment, the most notable changes were found in the effector CD4^+^ T-cell pool.

### Under treatment with tacrolimus, MMF and steroids, renal transplant recipients develop a memory-like B-cell phenotype

**Figure 3:**
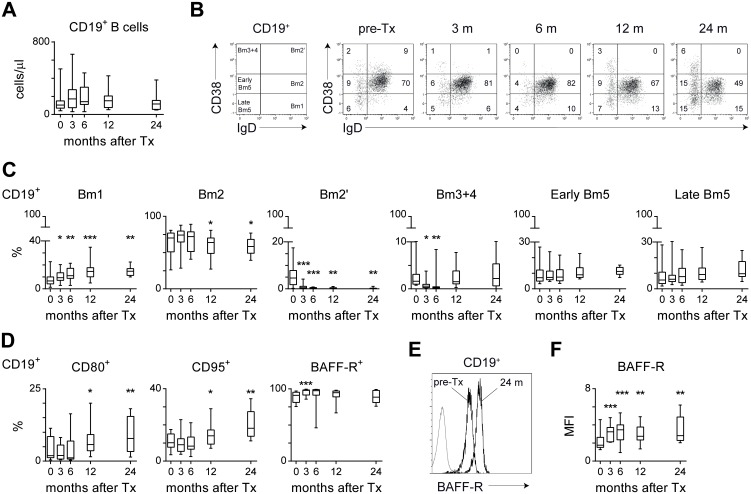
Longitudinal analysis of circulating B cells in renal transplant recipients after treatment with tacrolimus, MMF and steroids. (A) Shown are the absolute numbers of CD19^+^ B cells of 14 triple immunosuppression-treated patients before transplantation (t = 0) and at 3, 6, 12, and 24 months after transplantation (n = 10 at 24 m). (B) Representative dot plots for a renal transplant recipient over time using the Bm1-Bm5 classification: Bm1 (IgD^+^CD38^−^), Bm2 (IgD^+^CD38^+^), Bm2’ (IgD^+^CD38^++^), Bm3+4 (IgD^−^CD38^++^), Early Bm5 (IgD^−^CD38^+^) and Late Bm5 (IgD^−^CD38^−^) cells within the CD19^+^ B-cell population. (C) Shown are the percentages of the different B-cell subsets using the Bm1-Bm5 classification over time. (D) Shown are the percentages of CD80^+^, CD95^+^, and BAFF-receptor^+^ (BAFF-R) cells within the CD19^+^ B-cell population. (E) Overlay plot of the BAFF-R expression (MFI: median fluorescence intensity) within the CD19^+^ B-cell population of one patient before transplantation (pre-Tx) and 24 months (24 m) after transplantation under treatment with tacrolimus, MMF and steroids. Gray line shows unstained cells. (F) Summary graph showing the BAFF-R MFI of 14 triple immunosuppression-treated patients before over time (n = 10 at 24 m). Results are shown as box plots displaying the median, 25^th^ and 75^th^ percentiles as the box, and the 5^th^ and 95^th^ percentiles as whiskers. Significant differences are indicated compared to pre-transplant (pre-Tx; t = 0) levels: *P<0.05, **P<0.01, ***P<0.001.

During immunosuppressive treatment, the absolute B-cell numbers in peripheral blood were comparable to pre-transplant levels up to 24 months after transplantation (Figure 3A). To define whether the composition of the peripheral B-cell compartment was affected, we characterized CD19^+^ B cells using the Bm1-Bm5 classification [17]. Compared to pre-transplant levels, the percentages of naive Bm2 (IgD^+^CD38^+^) and transitional Bm2’ (IgD^+^CD38^++^) CD19^+^ cells were lower, and the percentage of late memory Bm5 (IgD^−^CD38^−^) cells was higher after transplantation (Figure 3B–C). This shift to a more memory-like phenotype was accompanied by an increase in the percentage of virgin naive Bm1 cells, probably needed for B-cell renewal. Accordingly, using an alternative terminology for B cells, the percentages of transitional CD24^++^CD38^++^, mature CD24^+^CD38^+^, and naive IgD^+^CD27^−^ B cells were decreased, while there was a relative increase in CD24^++^CD38^−^ memory and IgD^−^CD27^+^ switched memory B cells (Figure S2). At 12 and 24 months after transplantation, there was a higher percentage of CD80^+^ and CD95^+^ B cells as compared to before transplantation, supporting the more complete differentiation towards memory cells within the B-cell compartment (Figure 3D). The majority of B cells remained positive for B-cell activating factor receptor (BAFF-R) after transplantation. Interestingly, the expression level of BAFF-R increased after transplantation, as documented by an increase in median fluorescence intensity (MFI; Figure 3E–F). Overall, renal transplant recipients treated with tacrolimus, MMF and steroids developed a more memory-like B-cell phenotype.

### A single dose of RTX results in a long lasting B-cell depletion in peripheral blood without affecting the T-cell compartment

**Figure 4:**
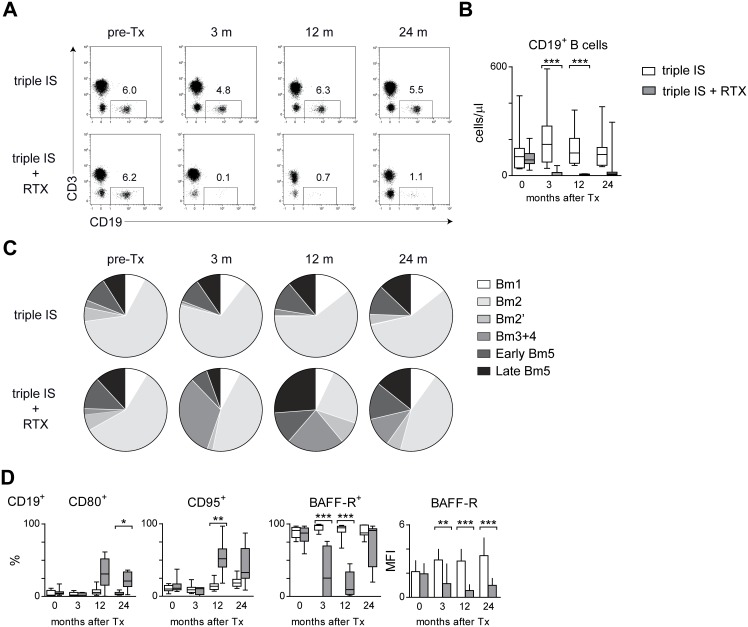
Longitudinal analysis of circulating B cells in renal transplant recipients after treatment with tacrolimus, MMF and steroids, and a single dose of rituximab (RTX) during transplant surgery. (A) Representative dot plots of CD19^+^ B cells within the CD45^+^ lymphocyte population for a RTX-treated and a triple immunosuppression (IS)-treated patient before transplantation (pre-Tx) and at 3, 12, and 24 months after transplantation. (B) Shown are the absolute numbers of CD19^+^ B cells for RTX-(gray, n = 12) and triple IS-treated (white, n = 14) patients before transplantation (t = 0) and up to 24 months after transplantation (n = 10 and n = 9 at t = 24 m, respectively). (C) Pie charts depicting the distribution the different B cells subsets over time using the Bm1-Bm5 classification as depicted in Figure 3B: Bm1 (IgD^+^CD38^−^), Bm2 (IgD^+^CD38^+^), Bm2’ (IgD^+^CD38^++^), Bm3+4 (IgD^−^CD38^++^), Early Bm5 (IgD^−^CD38^+^) and Late Bm5 (IgD^−^CD38^−^) cells within the CD19^+^ B-cell population. Data are represented as means of 14 triple IS+RTX-treated and 12 IS-treated patients before transplantation (pre-Tx) and at 3, 12, and 24 months after transplantation (n = 10 and n = 9 at t = 24 m, respectively). (D) Shown are the percentages of CD80^+^, CD95^+^ and BAFF-R^+^ cells within the CD19^+^ B-cell population for RTX- (gray, n = 12) and triple IS-treated (white, n = 14) patients before transplantation (t = 0) and up to 24 months after transplantation. Results are shown as box plots displaying the median, 25^th^ and 75^th^ percentiles as the box, and the 5^th^ and 95^th^ percentiles as whiskers. Significant differences are indicated by asterisks: **P<0.01.

The participation of renal transplant recipients in a randomized double-blind placebo-controlled study evaluating the efficacy and safety of RTX when added to triple drug immunosuppression gave us the opportunity to study the effects of additional B-cell depletion on the phenotype and function of T and B cells. RTX treatment resulted in a nearly complete depletion of B cells from the peripheral lymphocyte population up to 12 months after transplantation. Remarkably, the B-cell depletion after a single dose of RTX was long lasting. The absolute numbers of B cells remained quite low at 24 months after RTX treatment with a median of 9.7 B cells/µl (range 3.0–294.8) compared to a median of 116.4 B cells/µl (range 49.7–379.7) in patients not treated with RTX (Figure 4A–B). Interestingly, at 12 months the percentage of Bm2’ (IgD^++^CD38^++^) and Bm3+4 (IgD^−^CD38^++^) cells was significantly higher in RTX-treated patients, while the percentages of Bm1 (IgD^+^CD38^−^) and Bm2 (IgD^+^CD38^+^) cells were lower in RTX-treated patients. (Figure 4C). Accordingly, the percentages of CD24^++^CD38^++^ transitional and IgD^−^CD27^+^ switched memory B cells were higher in RTX-treated patients, while the percentages of IgD^+^CD27^−^ naïve B cells were lower in RTX-treated patients (Figure S3A). Fitting with the relative increase of Bm3+4 (IgD^−^CD38^++^) cells, there was an increase in the percentage of CD80^+^ and CD95^+^ B cells at 12 and/or 24 months after transplantation (Figure 4D). Remarkably, the percentage of BAFF-R^+^ B cells was lower in RTX-treated patients at 3 and 12 months after transplantation. In addition, the BAFF-R expression (MFI) on B cells of RTX-treated patients was lower up to 24 months after transplantation (Figure 4D).

Next, we analyzed whether the long-lasting B-cell depletion with a relative increase of transitional B cells resulted in changes in the T-cell compartment. There was no significant effect of RTX treatment on the absolute numbers and percentages of CD4^+^ and CD8^+^ T cells or on the subset distribution and phenotype of these cells (Figure S3B). In addition, *in vivo* B-cell depletion with RTX had no effect on the production of IL-2, IL-4, IL-17, IFNγ and TNFα by *ex vivo* stimulated T cells (Figure S3C).

## Discussion

Despite the extensive clinical experience with currently used immunosuppressive drug regimens, there are limited data available regarding their effects on the peripheral lymphocyte compartment after kidney transplantation. One study describes the effects of cyclosporine, MMF, steroids, and anti-CD25 monoclonal antibody therapy on T and B cells of mainly CMV seropositive renal transplant recipients at 6, 24, and 60 months after transplantation [18]. This therapy resulted in an increased percentage of CD4^+^CD25^+^ T_REGS_ and CD27^+^ memory B cells in renal transplant recipients compared to healthy donors [18], but the data were not compared with pre-transplant levels. In contrast, we performed a longitudinal analysis of T- and B-cell phenotype and function in CMV seronegative patients who received a kidney from a CMV seronegative donor and did not experience a rejection episode up to 24 months after transplantation. In this homogeneous patient population, not affected by major immunological events, we showed that treatment with the combination of tacrolimus, MMF and steroids had no effects on the total number of T and B cells. Nevertheless, these patients had a higher proportion of central memory CD4^+^ and CD8^+^ T cells at 3 months after transplantation compared to pre-transplant levels. Interestingly, the triple drug immunosuppression resulted in a shift toward a more memory-like phenotype in the B-cell population. Addition of a single dose of RTX resulted not only in a long-lasting B-cell depletion, but also in a higher percentage of transitional B cells upon B-cell recovery at 12 months post-transplant. The additional RTX treatment had no effect on the T-cell phenotype.

Although tacrolimus, MMF, and steroids mainly target T-cell activation, proliferation, and differentiation [3], [19], we found that treatment with a combination of tacrolimus, MMF, and steroids, induced only marginal changes in the peripheral T-cell phenotype. These changes were mainly present within the first 6 months after transplantation, which suggests a role for MMF, as this drug was discontinued at 6 months after transplantation. *Ex vivo*, the T cells collected from patients treated with triple immunosuppressive therapy were functional, suggesting that they are only suppressed when the drug is present (during treatment). In addition, we found that the ratio between CD4^+^ central memory and T_REGS_ was increased under triple drug immunosuppressive therapy. Concomitantly, we observed a relative increase of CXCR3^+^ and CCR6^+^ CD4^+^ T cells, chemokine receptors associated with memory or activated Th1 and Th17 cells, respectively. This expression enables them to migrate toward inflammatory sites that express their cognate chemokines [20], such as observed in the graft during rejection [21] and on activated human primary tubular epithelial cells [22].

With respect to B cells, mycophenolic acid, but not tacrolimus, has been shown to inhibit the proliferation and immunoglobulin production *in vitro* [23]. However, in patients with systemic lupus erythematosus who were treated with MMF, the number and phenotype of B cells were similar to that in controls without immunosuppressive therapy [24]. In our cohort, discontinuation of MMF at 6 months after transplantation resulted in a relative increase of virgin naive Bm1 cells, while naive Bm2 cells were decreased compared to pre-transplant levels. Transitional Bm2 cells remained low up to 24 months after transplantation, suggesting that their development is mainly suppressed by treatment with tacrolimus and/or steroids. Finally, following the discontinuation of MMF, the percentage of memory B cells became comparable to levels before transplantation. Steroids were also found to have clear effects on B cells; *ex vivo* immunoglobulin production by PBMC was decreased during treatment with a high dose of prednisolone (60 mg) while a lower dose (30 mg) resulted in an increased production after stimulation [25]. Others have described that steroids have an effect on B-cell activation, while proliferation and activation are less affected [26]. Under combined treatment with tacrolimus, MMF, and steroids, our renal transplant recipients had a more memory-like B-cell phenotype compared to before transplantation. This relative increase of memory B cells was also found in a patient cohort treated with cyclosporine, MMF, steroids, and an anti-CD25 monoclonal antibody [18]. The observed memory-like B-cell phenotype was accompanied by an increased percentage of CD80^+^ and CD95^+^ B cells, which may be explained by the preferential expression of these molecules on memory-like B cells [17].

Treatment with RTX provides a highly efficient means for the (temporary) depletion of B cells, with potential suppression of B cell-associated anti-graft responses. Adding a single dose of RTX to the combination of tacrolimus, MMF, and steroids in our patients indeed resulted in a long lasting B-cell depletion in peripheral blood. A remarkable characteristic of the returning B cells was a decreased expression of the receptor for B-cell activating factor (BAFF), an essential growth factor for B cells [27]. The decreased percentage of BAFF-R^+^ B cells after RTX treatment, which was also found in patients with rheumatoid arthritis [28] may be due to a relative increase of memory-like B cells, which have lower or no BAFF-R expression [27]. In addition, treatment with B-cell depleting agents has previously been shown to elevate BAFF levels [29]–[31], and increased BAFF levels in turn were inversely correlated with BAFF-R expression during B-cell repopulation [30], [32]. Another interesting observation was an increase in the percentage of transitional B cells at 12 months after treatment with RTX compared to triple immunosuppression therapy alone. Interestingly, BAFF-R deficiency in patients with common variable immunodeficiency (CVID) was associated with B-cell lymphopenia and a relative increase in the number of transitional B cells [33]. Taken together, an increase in BAFF level, reduced BAFF-R expression, and an increase in the proportion of transitional B cells appear to be interrelated phenomena which are associated with RTX treatment.

Upon activation, B cells are able to proliferate, produce various cytokines and process antigen for presentation to T cells [34]–[36]. Previously, we showed that *in vitro* RTX treatment can affect B-cell phenotype and function, resulting in an altered outcome of B-T-cell interaction upon stimulation [37]. However, in contrast, we did not observe any changes in the T-cell compartment in our patients treated with RTX. In another *ex vivo* study, we neither were able to show that RTX influenced the production of IL-17 and other monocyte- and T-cell-derived cytokines by PBMCs [38]. It should be noted however that we only analyzed peripheral blood T cells. From a previous study in renal transplant patients, we know that a single dose of RTX leads a to nearly complete B-cell depletion in peripheral blood, but not in secondary lymphoid organs, and that these remaining B cells have different functional capacities [39]. From our current data, it seems that this population of mostly memory type B cells residing in lymphoid organs does not noticeably affect the peripheral blood T-cell compartment as compared to transplant recipients on triple immunosuppression without RTX. Interestingly, several studies on patients with autoimmune disease revealed that the T-cell compartment was affected upon RTX treatment [12], [40], [41]. However, in most of these patients the cumulative dose of RTX was higher than in our patients, who received only a single, relatively low dose [40].

In summary, we have demonstrated that treatment of renal transplant recipients with tacrolimus, MMF and steroids leads to alterations in the T- and B-cell compartments. This detailed longitudinal analysis provides more insight into the immune status of renal transplant recipients with stable graft function and may be used as a reference in the monitoring of renal transplant patients.

## Supporting Information

Figure S1 **Patient selection for the side study of the clinical trial. CMV, Cytomegalovirus; D, donor; R, recipient.**
(EPS)

Figure S2 **Longitudinal analysis of circulating B cells in renal transplant recipients after treatment with tacrolimus, MMF and steroids.** Shown are the percentages of transitional CD24^++^CD38^++^, mature CD24^+^CD38^+^, memory CD24^++^CD38^−^, naive IgD^+^CD27^−^ and switched memory IgD^+^CD27^−^ B cells within the CD19^+^ B-cell population. Results are shown as box plots displaying the median, 25^th^ and 75^th^ percentiles as the box, and the 5^th^ and 95^th^ percentiles as whiskers. Significant differences are indicated compared to pre-transplant (t = 0) levels: *P<0.05, **P<0.01, ***P<0.001.
(EPS)

Figure S3 **Longitudinal analysis of circulating B and T cells in renal transplant recipients after treatment with tacrolimus, MMF and steroids, and a single dose of rituximab (RTX) during transplant surgery.** (A) Shown are the percentages of transitional CD24^++^CD38^++^, naive IgD^+^CD27^−^ and switched memory IgD^+^CD27^−^ B cells within the CD19^+^ B-cell population for RTX- (gray, n = 14) and triple IS-treated (white, n = 12) patients before transplantation (t = 0) and up to 24 months after transplantation (n = 10 and n = 9 at t = 24 m, respectively). (B) The percentage of regulatory T cells (T_REGS_; CD25^hi^CD127^lo^), central memory (T_CM_; CD27^+^CD45RO^+^), effector memory (T_EM_; CD27^−^CD45RO^+^), CXCR3^+^ and CCR6^+^ within the CD4^+^ T-cell population in RTX- (gray, n = 12) and triple IS-treated (white, n = 14) patients before transplantation (t = 0) and up to 24 months after transplantation (n = 10 and n = 9 at t = 24 m, respectively). (C) Peripheral blood mononuclear cells (PBMCs) were stimulated for 4 hours in the presence of PMA, ionomycin and Brefeldin A. Shown are the percentages IL-2, IL-4, IL-17, IFNγ or TNFα-producing cells within the CD4^+^ T-cell population in RTX- (gray, n = 12) and triple IS-treated (white, n = 14) patients before transplantation (t = 0) and up to 24 months after transplantation (n = 10 and n = 9 at t = 24 m, respectively). Results are shown as box plots displaying the median, 25^th^ and 75^th^ percentiles as the box, and the 5^th^ and 95^th^ percentiles as whiskers. Significant differences are indicated by asterisks: **P<0.01.
(EPS)
